# Biomechanical perspectives on image-based hip fracture risk assessment: advances and challenges

**DOI:** 10.3389/fendo.2025.1538460

**Published:** 2025-03-04

**Authors:** Yunhua Luo

**Affiliations:** ^1^ Department of Mechanical Engineering, University of Manitoba, Winnipeg, MB, Canada; ^2^ Department of Biomedical Engineering (Graduate Program), University of Manitoba, Winnipeg, MB, Canada

**Keywords:** hip fracture, risk assessment, DXA, QCT, bone strength, fall-induced impact force

## Abstract

Hip fractures pose a significant health challenge, particularly in aging populations, leading to substantial morbidity and economic burden. Most hip fractures result from a combination of osteoporosis and falls. Accurate assessment of hip fracture risk is essential for identifying high-risk individuals and implementing effective preventive strategies. Current clinical tools, such as the Fracture Risk Assessment Tool (FRAX), primarily rely on statistical models of clinical risk factors derived from large population studies. However, these tools often lack specificity in capturing the individual biomechanical factors that directly influence fracture susceptibility. Consequently, image-based biomechanical approaches, primarily leveraging dual-energy X-ray absorptiometry (DXA) and quantitative computed tomography (QCT), have garnered attention for their potential to provide a more precise evaluation of bone strength and the impact forces involved in falls, thereby enhancing risk prediction accuracy. Biomechanical approaches rely on two fundamental components: assessing bone strength and predicting fall-induced impact forces. While significant advancements have been made in image-based finite element (FE) modeling for bone strength analysis and dynamic simulations of fall-induced impact forces, substantial challenges remain. In this review, we examine recent progress in these areas and highlight the key challenges that must be addressed to advance the field and improve fracture risk prediction.

## 1 Introduction

Hip fractures are a significant health concern, particularly among older adults, who often have a high prevalence of osteoporosis, contributing to substantial morbidity, mortality, and healthcare costs worldwide (1–3). In 2019, there were 178 million new fractures globally, marking a 33.4% increase since 1990, partly driven by population aging (2, 3). Hip fractures constituted a significant proportion of these cases. Projections indicate that the number of hip fractures will nearly double by 2050, underscoring the urgency for effective fracture risk assessment to identify high-risk individuals and implement preventive measures (4, 5). Accurate assessment of hip fracture risk is crucial, as it enables targeted interventions and support, thereby reducing the burden of these fractures (6).

The current clinical approach for diagnosing hip fracture risk and treating pre-fracture conditions relies primarily on risk factors such as bone mineral density (BMD) and population-based statistical models (7). Although low BMD is widely regarded as a key biomarker for bone fractures, this approach has significant limitations. Studies indicate that approximately 50% of fractures occur in individuals with BMD values above the established threshold (8, 9). BMD also serves as the primary target for many treatment options, particularly those aimed at osteoporosis (10, 11). FRAX is one of the most widely used tools globally to estimate the 10-year probability of hip fractures and other major osteoporotic fractures (12–15). It incorporates several key risk factors, including age, gender, BMD at the femoral neck, prior fractures, parental history of hip fractures, smoking status, alcohol consumption, glucocorticoid use, and rheumatoid arthritis. The predictive accuracy of FRAX has been reported as moderate (16, 17), with area under the receiver operating characteristic (ROC) curve (AUC) values ranging from 0.70 to 0.75 for hip fracture prediction. The tool tends to underestimate fracture risk in certain populations, such as those with frequent falls or advanced age, where fall risk is not fully incorporated (14, 18, 19). The primary limitation of the current tools lies in their reliance on statistical modeling of risk factors. These tools predict fracture risk by identifying broad population-level trends and applying them to individual cases (20).

To improve the accuracy of hip fracture risk assessments, there is a pressing need to develop biomechanical models (21). Image-based biomechanical approaches are theoretically more reliable and accurate than statistical models derived from clinical risk factors because they directly assess the mechanical properties of bone and the forces contributing to fractures (22, 23). Unlike statistical models, which rely on population-level data and indirect associations, biomechanical approaches evaluate individual-specific factors such as bone strength, geometry, and microstructure. These methods utilize advanced imaging techniques, such as high-resolution CT and finite element (FE) modeling, to simulate the mechanical response of bones to applied forces, providing a direct measurement of fracture risk. Furthermore, image-based dynamic simulations can model fall-induced impact forces by analyzing body kinematics, fall trajectories, and surface interactions (24, 25). These simulations allow for a detailed assessment of the magnitude, direction, and distribution of impact forces during a fall (25), which are critical in determining fracture risk. By integrating subject-specific bone properties with dynamic fall scenarios (26), biomechanical approaches can provide a comprehensive and personalized evaluation of fracture risk, addressing limitations in clinical tools that overlook the interplay between bone strength and fall mechanics. This capability highlights their potential to significantly enhance fracture risk assessment and prevention strategies.

Substantial progress has been made in developing image-based biomechanical models for predicting hip fracture risk. However, significant challenges remain, which must be addressed before these biomechanical models can be integrated into clinical practice. This review extensively examines recent advancements and discusses the key challenges that need resolution. The layout of the remainder of this paper is as follows: Section 2 outlines the framework of image-based biomechanical approaches; Section 3 reviews the progress and challenges in image-based finite element modeling of bone strength; Section 4 explores the advancements and remaining obstacles in image-based dynamic simulation of falls; and Section 5 concludes the review with proposals for future research directions.

## 2 Image-based biomechanical approach to assess hip fracture risk

**Figure 1:**
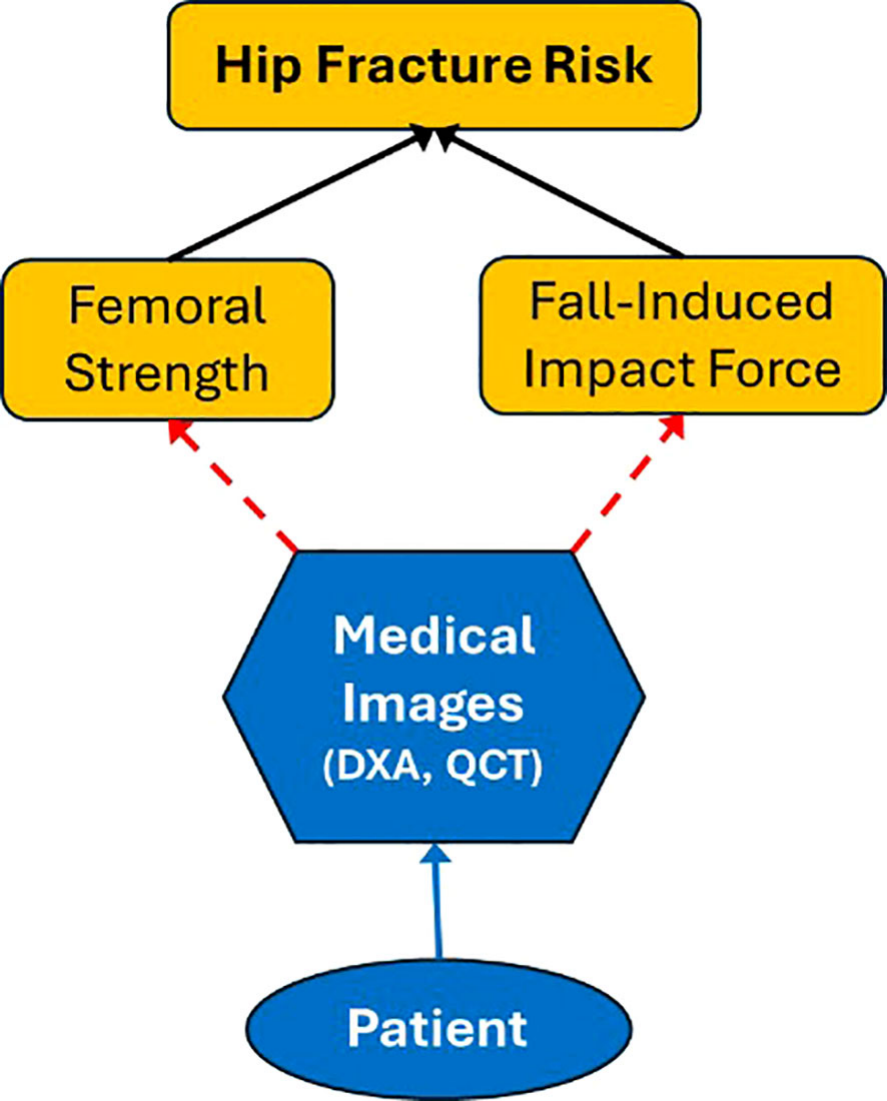
Image-based biomechanical approach to assess hip fracture risk.

Based on engineering material mechanics, hip fracture is determined by two key variables (**Figure 1**): femoral strength and the force applied to the hip, both of which are subject-specific. Femoral strength refers to the maximum force the femur can withstand before fracturing and is primarily determined by the bone’s material composition—such as inorganic minerals, organic proteins, and water—along with its macroscopic geometry and microstructural integrity. Since the majority of hip fractures result from falls (27–30), the impact force generated during a fall from standing height is considered in assessing hip fracture risk. This force is influenced by variables such as body height, body mass, and fall orientation and can vary significantly depending on the dynamics of the fall and the compliance properties of the impacted surface. When the fall-induced force exceeds femoral strength, a hip fracture occurs. Accurately determining femoral strength, fall-induced impact force, and their interplay is essential for developing precise and predictive models of fracture risk, enabling more effective prevention and individualized treatment strategies.

Given the necessity for non-invasive approaches in assessing hip fracture risk, determining both femoral strength and fall-induced impact force must be conducted safely and without invasive procedures. Medical imaging offers an essential solution to this challenge, as illustrated in **Figure 1**. Advanced imaging technologies, such as dual-energy X-ray absorptiometry (DXA) and quantitative computed tomography (QCT), enable subject-specific assessment of bone structure, geometry, and tissue composition within the body. These imaging modalities provide critical data on bone mineral density, and material composition, which are essential for estimating femoral strength. Information about trabecular architecture can be partially inferred from QCT data, as it provides 3D volumetric imaging capable of analyzing parameters such as trabecular thickness, separation, and number. However, DXA, being a 2D imaging modality, lacks the resolution to capture detailed trabecular architecture. For more precise insights into individual trabecular microstructure, higher-resolution imaging modalities such as micro-CT or HR-pQCT are required, although these are typically limited to *in vitro* studies or extremities *in vivo*. Furthermore, imaging can capture patient-specific anatomical and kinetic properties, which can then be used in dynamic simulations to predict fall-induced impact forces. The integration of imaging data into biomechanical models ensures a personalized and accurate evaluation of fracture risk.

Significant advances have been made in the development of image-based finite element (FE) modeling for predicting femoral strength and dynamics simulations for analyzing fall-induced impact forces. While these advancements offer promising opportunities to assess subject-specific fracture risk more accurately, challenges and obstacles remain. The following sections provide a detailed review of these advancements, highlighting the progress achieved and the critical barriers that must be addressed to facilitate their integration into clinical practice.

## 3 Image-based finite element modeling of bone strength

To construct a finite element (FE) model of the femur for determining its strength, several key pieces of information are required. First, accurate geometry of the femur is essential, typically derived from high-resolution medical imaging modalities such as computed tomography (CT). These images provide detailed spatial data that allow for the reconstruction of the femur’s shape and structural features, including cortical thickness, trabecular architecture, and overall bone dimensions. Second, the material properties of the bone must be specified, including the elastic modulus, yield strength, and density of both cortical and trabecular bone. These properties are often determined from CT-derived Hounsfield units, which can be mapped to bone density and subsequently used to estimate the material properties. Additionally, boundary conditions and loading scenarios must be defined to replicate physiological or fall-related forces acting on the femur, such as compressive loads during standing or oblique forces during a fall. Together, these inputs enable the FE model to simulate stress and strain distributions within the femur and predict its failure point under applied loads.

While DXA and QCT are the primary imaging modalities discussed in this paper due to their clinical relevance for biomechanical modeling, other advanced imaging technologies also hold promise. High-resolution peripheral QCT (HR-pQCT) offers detailed insights into bone microarchitecture but is limited to extremities due to its field of view. Dual-energy CT (DECT) enables improved material characterization by distinguishing between bone mineral density and other components, such as collagen and water. Magnetic resonance imaging (MRI) can provide complementary information on bone marrow composition and trabecular structure but lacks the spatial resolution necessary for finite element modeling of bone strength. Although these techniques have significant potential, their high cost, limited availability, and practical constraints currently limit their widespread application in hip fracture risk assessment.

Numerous finite element (FE) models have been developed for the femur, with most falling into two primary categories: those based on dual-energy X-ray absorptiometry (DXA) and those based on quantitative computed tomography (QCT).

### 3.1 DXA-based finite element models

**Figure 2:**
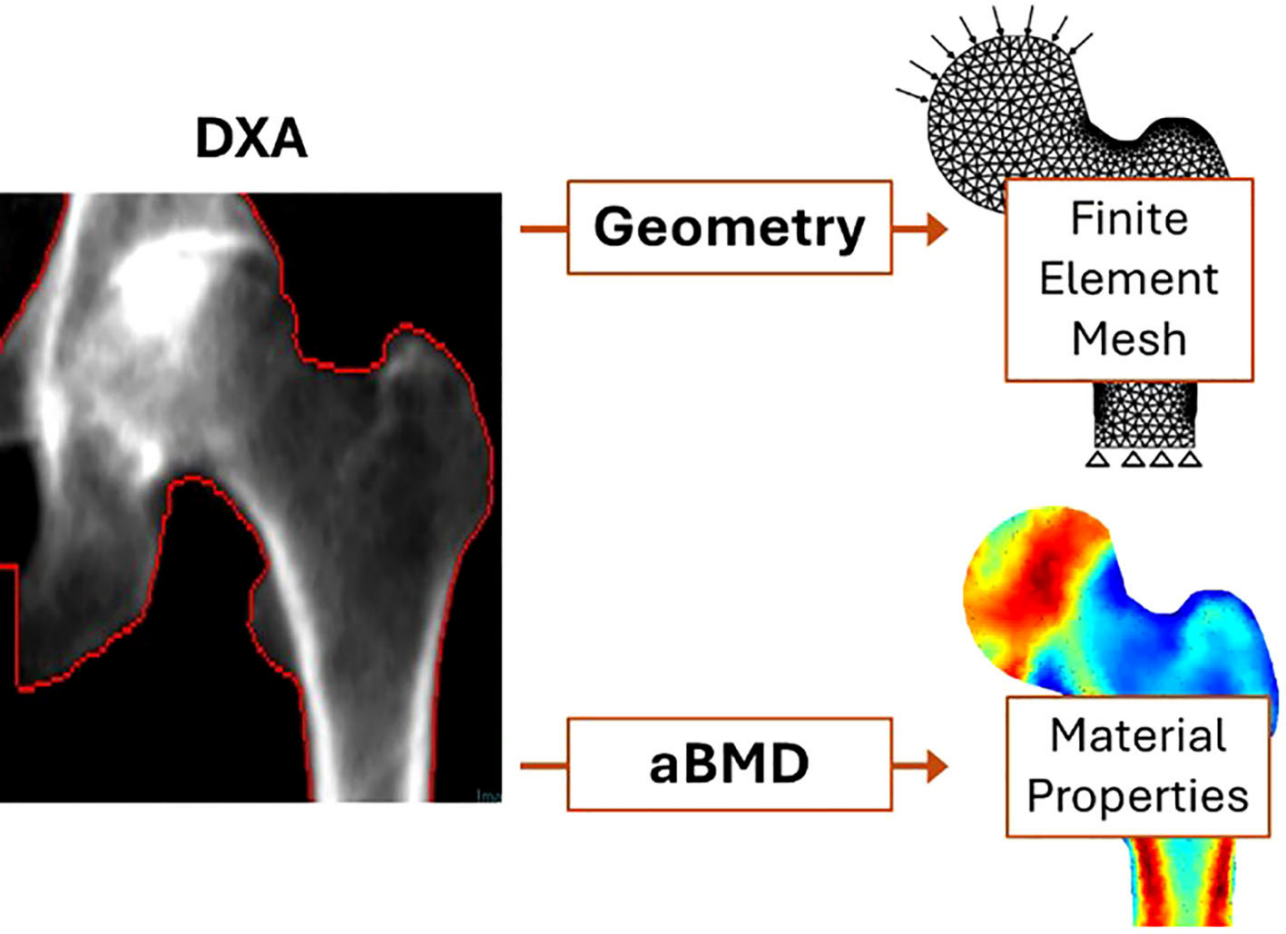
DXA-based finite element modeling of femoral strength [modified from (34, 35)].

DXA-based FE models are particularly attractive due to the merits of DXA over QCT, including lower cost, wider availability, and reduced radiation exposure. These models leverage two-dimensional (2D) DXA images to estimate femoral strength and fracture risk by incorporating simplified assumptions about bone geometry and material properties, as illustrated in **Figure 2**. First, a plane stress model (31) or engineering beam model (32) is adopted, representing the femur by projecting all the bone material along the DXA scanning direction, thereby reducing the complex 3D geometry of the femur to a simplified 2D model with uniform thickness. Second, the areal bone mineral density (aBMD) derived from DXA is correlated with key material properties (33), such as bone elasticity and yield stress, enabling the estimation of bone strength in the medial-lateral plane.

In DXA-based finite element analyses, material models primarily assume linear elastic behavior due to the simplicity and computational efficiency required for clinical applicability (36, 37). For instance, the Young’s modulus is often estimated based on empirical relationships with areal bone mineral density (aBMD) (36, 37). Some studies incorporate piecewise linear models to account for yield points and post-yield behavior, though these are less common due to the limitations of 2D projections in capturing detailed material heterogeneity. Non-linear models, which consider failure criteria or plasticity, have been less frequently applied in DXA-based FE analyses due to the challenges in accurately representing complex bone material using 2D data (38). DXA-based finite element studies commonly employ simplified yet clinically relevant loading and boundary conditions to simulate scenarios associated with hip fractures. The most frequently used loading scenario involves a sideways fall, which reflects the most common fall mechanism leading to hip fractures in older adults. In these simulations, the femoral head is typically subjected to oblique forces, representing the impact of the greater trochanter against the ground during a fall. These assumptions and correlations strike a practical balance between prediction accuracy and simplicity, positioning DXA-based FE models as a promising patient-specific tool for assessing hip fracture risk in clinical settings.

The development and improvement of DXA-based finite element models for hip fracture risk assessment have significantly advanced, driven by the need for more accurate and individualized predictions of bone strength and fracture risk. Early studies demonstrated the feasibility of integrating finite element analysis (FEA) with DXA imaging to estimate femoral strength (31, 34, 39). Luo et al. (35) investigated the precision of DXA-based finite element models, identifying body positioning during DXA scanning as a critical factor influencing model accuracy. Further advancements focused on automation and clinical applicability, as illustrated by Luo et al. (40) and Yang et al. (41), who developed fully automated DXA-based FEA tools that not only stratified fracture risk more effectively than femoral neck bone mineral density (BMD) but also streamlined workflows for routine clinical use. Validation efforts, such as those by Dall’Ara et al. (42), confirmed the accuracy of DXA-based FEA models against experimental data, reinforcing their reliability. Simplified 2D FEA models derived from DXA images were also validated against more complex 3D models by Terzini et al. (38), highlighting their practicality with reasonable predictive accuracy. These continuous improvements have established DXA-based FEA as a robust and clinically viable approach to addressing the limitations of traditional BMD-focused fracture risk assessments. DXA-based finite element models are increasingly being utilized for hip fracture risk assessment. Yang et al. (43) demonstrated the effectiveness of this approach in the Osteoporotic Fractures in Men (MrOS) study, where femoral strength estimates derived from FEA showed a strong association with incident fractures. Sarvi and Luo (44) investigated sex differences in hip fracture risk using biomechanical modeling and identified significant distinctions that traditional BMD measurements failed to capture. Additionally, Ferdous et al. (31) underscored the value of patient-specific FEA models in evaluating individualized fracture risk, further highlighting the adaptability and clinical potential of this technique. In addition to risk assessment, DXA-based FE models have been used to monitor the effectiveness of osteoporosis treatments. Mochizuki et al. (45) employed DXA-based hip structural analysis to evaluate changes in bone strength during teriparatide treatment, demonstrating significant improvements in femoral strength over 24 months.

Despite their advantages, DXA-based FE models have several limitations. DXA only provides 2D projections of the femur, which limits the model’s ability to capture the 3D geometry and microstructural details essential for accurate stress and strain predictions. DXA-based FE models often rely on oversimplified assumptions about the relationship between aBMD and bone material properties. These assumptions may overlook variations in the spatial distribution of bone mass, including differences in cortical and cancellous component densities, which are critical for capturing the anisotropic nature of femoral strength. Additionally, the 2D nature of DXA imaging restricts its capacity to evaluate trabecular architecture and cortical porosity, both of which are essential determinants of bone strength and fracture risk. Anatomical geometry reconstruction from DXA images models the entire femur as a single entity (46, 47), assigning subject-specific material properties based on areal bone mineral density (aBMD) values derived from DXA images. This method simplifies the geometry and computational requirements but inherently lacks the ability to distinguish between cortical and trabecular compartments, which are critical for accurately capturing the heterogeneity of bone properties. DXA-based FE models often use simplified loading scenarios to estimate femoral strength, which may not accurately represent the complex, multidirectional forces experienced during real-world falls. DXA-based FE models primarily reflect changes in BMD, making them less sensitive to other critical factors, such as improvements in bone collagen quality and the integrity of collagen crosslinks (48, 49), which play a significant role in bone strength and may result from osteoporosis treatments. Variations in DXA scanner calibration and software algorithms (50) can introduce inconsistencies in BMD measurements, affecting the reproducibility of FE model predictions.

### 3.2 QCT-based finite element models

**Figure 3:**
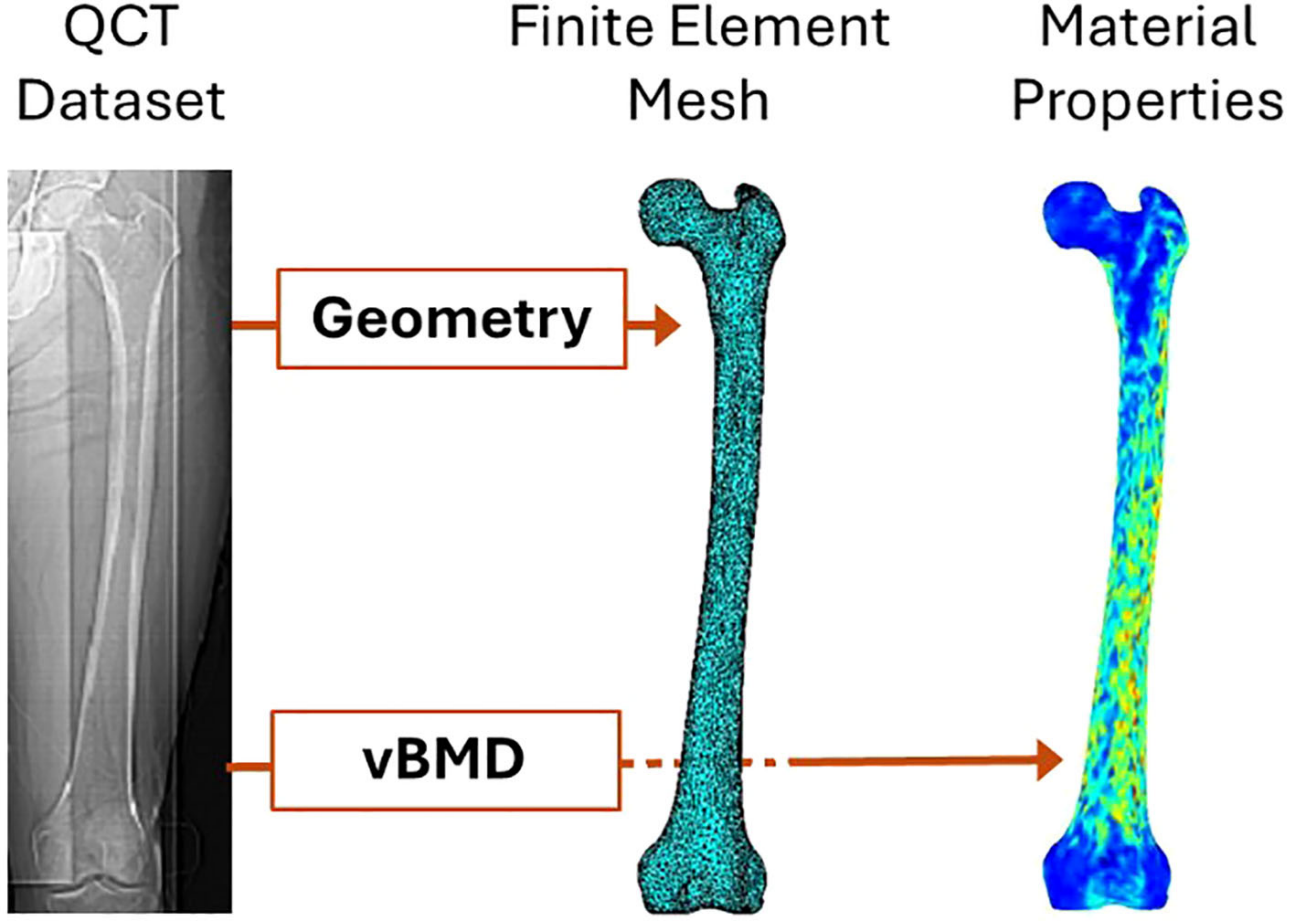
QCT-based finite element modeling of femoral strength [modified from (55)].

QCT-based finite element models are constructed from three-dimensional data acquired through quantitative computed tomography (QCT). While the process of creating QCT-based finite element models shares similarities with that of DXA-based models, as illustrated in **Figure 3**, the key differences lie in the three-dimensional representation of femur geometry and the use of volumetric bone mineral density (vBMD) instead of areal BMD (51). The process begins with acquiring high-resolution QCT images of the femur. These datasets are segmented to differentiate bone tissue from surrounding structures, enabling the extraction of cortical and trabecular bone regions (52). The image data are then converted into 3D finite element meshes, typically composed of tetrahedral or hexahedral elements, to accurately approximate the femoral geometry (53). Tetrahedral elements are more versatile in conforming to complex geometries, making them suitable for irregular structures like the femur. In contrast, hexahedral elements offer higher accuracy and computational efficiency for simpler, structured geometries but are less adaptable to irregular shapes. The choice between the two depends on the trade-off between geometric fidelity and computational efficiency in the modeling process. Bone densities are obtained from QCT image intensities through calibration with phantoms, which provide reference values for converting Hounsfield units into equivalent bone density measures. Material properties are assigned based on the density values using empirical relationships that link density to Young’s modulus and other mechanical parameters (51, 54). Boundary and loading conditions are applied to simulate physiological or traumatic scenarios, such as normal gait or sideways falls (52). Finally, these models are solved using numerical methods to estimate stress, strain, and overall femoral strength (53). Overall femoral strength is typically defined as the maximum load the femur can withstand before failure, as determined by the finite element simulation. This definition depends on the material model used; for linear elastic models, it is based on yield stress, while for non-linear models, it may incorporate ultimate stress or fracture criteria. The choice of strength definition varies depending on the specific study objectives and modeling assumptions.

QCT-based finite element models have emerged as a robust tool for studying femoral strength and hip fracture risk. By integrating 3D imaging with advanced computational modeling, these models provide detailed insights into the mechanical behavior of the femur under different loading conditions. Below, we review key research applications of QCT-based FE models in this domain. QCT-based FE models have been widely used to evaluate the structural integrity of the femur under simulated loading conditions. Dragomir-Daescu et al. (56) developed robust models to predict femoral stiffness and fracture load during a sideways fall, demonstrating strong correlations with *in vitro* experimental data. Mirzaei et al. (57) applied QCT-based FE analysis to analyze strength and failure patterns in the human proximal femur, revealing critical mechanical insights that aid in fracture prediction. Dall’Ara et al. (58) validated nonlinear QCT-based FE models using *in vitro* human femur, showing their reliability across multiple experimental configurations. These studies highlight the utility of QCT-based FE models in quantifying femoral strength and identifying high-risk individuals.

QCT-based FE modeling has also been employed to study variations in femoral strength across different populations. Shen et al. (59) investigated the relationship between body mass index (BMI) and QCT-derived hip strength in older men, providing biomechanical explanations for the effects of BMI on fracture risk. Black et al. (60) conducted a large prospective study to assess the relationship between proximal femoral structure, as derived from QCT, and hip fracture risk in men, establishing the clinical relevance of QCT-based measurements. Faisal and Luo (55) examined differences in fracture risk between left and right femora using QCT-based FE models, identifying asymmetries that may inform individualized treatment strategies. Several studies have focused on evaluating hip fracture risk under specific mechanical or pathological conditions using QCT-based FE models. For example, Kheirollahi and Luo (61) used cross-sectional strain energy derived from QCT-based FE models to assess hip fracture risk, demonstrating the sensitivity of this method to variations in bone density and geometry. Carpenter et al. (62) emphasized the importance of fall orientation on femoral neck strength, showing that certain fall directions substantially increase fracture risk. Such studies underline the versatility of QCT-based FE models in replicating realistic fracture scenarios.

Traditional metrics for monitoring treatment effects typically include changes in areal bone mineral density (aBMD) as measured by DXA. Treatments such as bisphosphonates or anabolic agents like teriparatide are commonly assessed using these metrics. These methods focus on improving bone density and strength over time, offering a baseline for evaluating therapeutic outcomes. QCT-based FE models are employed to examine the contributions of cortical and trabecular compartments to overall femoral strength. Christiansen et al. (63) used these models to explore age-related changes in bone strength, showing how the cortical and trabecular components contribute differently to mechanical stability in men and women. These findings have enhanced the understanding of how age and sex influence fracture risk. QCT-based FE models have been applied to evaluate the impact of osteoporosis treatments and other clinical conditions on femoral strength. Engelke et al. (64) used these models to monitor regional changes in bone mineral density after ibandronate treatment, demonstrating how such treatments improve hip strength. Similarly, Black et al. (60) showed how QCT-based parameters could predict treatment outcomes more effectively than traditional metrics, emphasizing the potential of these models in clinical decision-making.

QCT-based finite element models provide a more detailed and robust approach than DXA-based models for assessing femoral strength and hip fracture risk. QCT offers greater detail compared to DXA by providing 3D volumetric imaging, allowing separate analysis of cortical and trabecular compartments. Additionally, QCT measures volumetric bone mineral density (vBMD), which is not influenced by bone size or projection errors, and enables assessment of bone geometry, microarchitecture, and material properties with higher spatial resolution. However, QCT-based models are not without limitations. A major challenge lies in the high radiation dose associated with QCT imaging, which restricts its routine clinical use, especially for longitudinal studies (65). Additionally, constructing and solving QCT-based finite element models require advanced computational resources and expertise, which can be a barrier to widespread adoption in clinical practice (56). Variability in imaging protocols and finite element modeling assumptions, such as mesh density and material property assignment, can introduce inconsistencies and limit reproducibility across studies (58, 63). Furthermore, the use of density-based material property assignment often oversimplifies bone’s heterogeneous and anisotropic mechanical behavior, potentially reducing the accuracy of predictions (57). Finally, these models generally do not account for dynamic biological processes, such as bone remodeling or microdamage accumulation, which are critical for understanding changes in bone strength over time (61). Addressing these limitations through advancements in imaging, modeling, and computational techniques is essential to enhance the clinical utility of QCT-based finite element models.

### 3.3 Challenges in image-based finite element modeling of femoral strength

Finite element modeling of femoral strength based on medical imaging, such as QCT or DXA, has advanced significantly in recent years, offering valuable insights into bone mechanics and fracture risk. However, despite these advancements, and alongside the limitations discussed in the previous subsections, several critical challenges persist, hindering the accuracy, reliability, and clinical utility of these models. One major issue lies in the challenge of accurately characterizing bone material properties, such as Young’s modulus, yield stress, and toughness, from medical images. Another significant challenge is capturing the anisotropic behavior of femoral strength, which varies with loading orientation and is influenced by the direction of impact forces during a fall. Bone anisotropy has been studied in vertebral bones (66), where transverse isotropy is modeled by scaling Young’s modulus according to directional properties. Application of a similar approach to the femur requires experimentally derived scaling factors. Addressing these complexities requires advancements in imaging technologies, image-based material characterization algorithms, and modeling techniques, as these elements are pivotal for enhancing the predictive accuracy and reliability of image-based finite element models.

#### 3.3.1 Image-based characterization of bone material properties

Accurately characterizing bone material properties, such as Young’s modulus, yield stress, and toughness, from DXA or QCT images remains a significant challenge due to the composite nature of bone. Bone is a hierarchical material composed of inorganic minerals (primarily hydroxyapatite), organic proteins (mostly collagen), and water. Each of these components contributes distinct mechanical properties to bone, and their interplay determines the overall strength and toughness of the tissue. However, medical imaging modalities like DXA and QCT are limited in their ability to quantify or assess the quality of these individual components, which hinders precise material characterization.

DXA and QCT provide information about bone density, which is a proxy for the amount of mineral content in bone. However, this metric alone does not capture variations in the organic matrix or water content, both of which critically influence mechanical properties. Studies have shown that the organic matrix, particularly collagen cross-linking, plays a pivotal role in bone toughness and resistance to fracture (67–69). Similarly, bound and free water in bone contribute to its viscoelastic and fatigue-resistant properties (70). Limited by their working principles, both DXA and QCT can measure only mineral density, while the characterization of organic proteins and water remains challenging with these imaging modalities. As a result, the contributions of organic proteins and water to bone strength, particularly toughness, are not accounted for in DXA- and QCT-based models (71, 72). Further complicating the issue is the heterogeneity of bone mineralization. The degree of mineralization varies across individuals and regions within the bone, affecting stiffness and brittleness. QCT-based finite element models often rely on empirical density-elasticity relationships derived from bone properties, which may not account for inter-individual variability in the inorganic-organic composition or regional differences within the same bone (54, 73). This limitation undermines the ability to predict mechanical properties accurately under diverse physiological or pathological conditions.

Another critical challenge lies in accurately determining the stress-strain curves for the individual components of bone, particularly minerals and proteins, which are dependent on the sub-compositions and sub-microstructure in the components. These curves are fundamental for understanding bone behavior under impact forces but are highly subject-dependent, adding complexity to their precise characterization. For instance, the mechanical behavior of hydroxyapatite, the primary mineral in bone, depends on its crystal size, orientation, and substitutional chemistry, all of which can vary significantly among individuals (74). Similarly, the organic matrix, predominantly composed of type I collagen, shows variability in structure and cross-linking patterns among individuals, directly influencing its mechanical response under load (75). Factors such as age, sex, ethnicity, and health status further modulate the quality and quantity of these bone components, resulting in significant differences in their mechanical properties (67). For example, aging reduces collagen quality while increasing mineral crystallinity, leading to stiffer but more brittle bones (76). Imaging modalities like QCT and DXA currently lack the capability to capture these subtle yet critical changes in bone composition and quality. Furthermore, the absence of standardized methods for characterizing these properties either *in vivo* or *ex vivo* complicates their integration into finite element models, underscoring a significant limitation in current biomechanical assessments.

Furthermore, the interaction between the inorganic and organic components introduces non-linearities that are not easily captured by existing imaging techniques. For example, the role of collagen in resisting crack propagation and maintaining post-yield behavior is critical for bone toughness, but current imaging modalities cannot quantify the functional quality of collagen or its integration with the mineral phase (49, 77). Advances in techniques like Raman spectroscopy and nanoindentation have provided insights into these interactions *in vitro*, but these are not yet translatable to clinical imaging settings. Raman spectroscopy, including methods like surface-enhanced Raman scattering (SERS) and tip-enhanced Raman scattering (TERS), offers detailed molecular information and high spatial resolution (78). Nanoindentation, on the other hand, allows for precise measurement of mechanical properties at the nanoscale (79). Despite their potential, these techniques face challenges in clinical translation due to issues like signal interference and the complexity of *in vivo* environments.

Addressing the challenges of characterizing bone material properties from medical images requires significant advancements in imaging technologies and computational modeling. Techniques that integrate imaging with compositional analysis, such as dual-energy CT (DECT) or high-resolution peripheral QCT (HR-pQCT), hold promise but remain in early stages of application (80). HR-pQCT is currently limited to extremities due to hardware and radiation constraints, making their use for larger regions like the proximal femur impractical. Empirical data from cadaveric studies could enhance finite element models, and future research could explore hybrid approaches combining high-resolution data with clinical imaging. Balancing radiation exposure with the need for detailed imaging is critical. Leveraging already-acquired clinical images for biomechanical modeling can reduce the need for additional scans. Standardizing imaging protocols in advance can further minimize radiation dose and costs while maintaining the necessary level of detail for accurate finite element analyses. Expanding our understanding of the material behavior of bone’s components and improving the resolution and functionality of medical imaging will be critical for advancing finite element models and their clinical utility (81).

#### 3.3.2 Anisotropy in bone mechanical properties and femoral strength

Anisotropy in bone mechanical properties refers to the variation in mechanical characteristics, such as Young’s modulus and ultimate stress, depending on the orientation of the bone test sample, even when taken from the same site. Similarly, anisotropy in femoral strength indicates that the maximum force the femur can sustain before fracturing varies with the direction of the applied force. This anisotropy arises from bone’s hierarchical structure and composition, including the alignment of collagen fibers, the distribution of hydroxyapatite crystals, and the trabecular architecture within the femoral head and neck (75, 82, 83). Cortical bone in the femoral shaft, for instance, is stiffer and stronger along the longitudinal axis, making it particularly effective at resisting axial loads during activities like walking and running (84). In contrast, the trabecular bone in the femoral head and neck features a highly complex, orientation-specific architecture designed to distribute stresses arising from multi-directional loading scenarios (85).

The majority of QCT-based finite element models employ simplified isotropic material assumptions for bone mechanical properties; however, they can still demonstrate the anisotropic nature of femoral strength due to the influence of bone geometry and heterogeneous material distribution. These models reveal that bone is more resistant to compression and tension in certain orientations while being more susceptible to shear forces in others (52, 86). Studies have shown that the femur’s ability to withstand impact forces is highly dependent on the direction and magnitude of the force applied during a fall (24, 87, 88). For example, sideways falls, which are the most common fall scenario in elderly individuals, generate impact forces that are poorly aligned with the femur’s primary axis of strength, significantly increasing the risk of fracture (24). Conversely, frontal or posterior falls may exert forces along directions that the femur is better adapted to withstand, reducing fracture risk (87, 88).

However, the isotropic models of bone mechanical properties inherently overlook the directional dependence of these properties, limiting their accuracy in simulating real-world loading conditions. To address this limitation, finite element models must incorporate anisotropic mechanical properties that reflect the true directional behavior of bone material. Achieving this level of precision requires advanced imaging and material characterization techniques, such as those capable of capturing collagen fiber orientation and mineral distribution, which are not yet widely accessible. This presents a significant barrier to advancing modeling accuracy and clinical applicability.

Current imaging modalities, such as QCT and DXA, are limited in their ability to comprehensively characterize bone composition, including inorganic minerals, organic proteins, and water, let alone provide detailed orientation-specific data on bone strength (89, 90). Incorporating composition- and microstructure-dependent mechanical properties and anisotropy into finite element models requires a deeper understanding of the hierarchical structure of bone, particularly the trabecular and cortical microstructures. Advanced imaging techniques, such as dual-energy computed tomography (DECT), offer promising avenues for distinguishing and quantifying bone components with greater specificity (91, 92). However, these techniques are still under development and face challenges such as resolution limitations and the accurate extraction of anisotropic mechanical properties. Overcoming these barriers is essential to achieving more precise and clinically relevant models for fracture risk assessment.

## 4 Image-based dynamics modeling of falls to predict impact forces

Falls are the leading cause of hip fractures, with over 95% of hip fractures attributed to falls from standing height (93). The forces generated during a fall frequently exceed the strength of the femur, resulting in fractures even in young, healthy individuals—let alone older adults, who often have compromised bone strength due to age-related changes or conditions like osteoporosis (94). However, only 2% of falls result in fractures (95, 96), highlighting the complex interplay between individual biomechanics, fall dynamics, and environmental factors. This low percentage underscores the importance of understanding how variables such as bone strength, fall-induced forces, body orientation during impact, and surface compliance collectively influence fracture outcomes. Fall experiments, even controlled fall testing, are neither ethical nor safe for elderly individuals. Image-based dynamics modeling offers a promising alternative for simulating falls and predicting impact forces by integrating subject-specific anatomical and biomechanical data derived from advanced medical imaging techniques.

This section explores the necessity and potential of subject-specific dynamics modeling for fall simulations to predict impact forces. It discusses the importance of incorporating whole-body imaging data, such as DXA or QCT, to create accurate models, reviews the progress made in simulating falls from standing height, and examines the challenges that must be addressed to replicate real-world fall scenarios. By leveraging these advancements, the goal is to improve fracture risk prediction and develop more effective prevention strategies.

### 4.1 Subject-specific dynamics modeling of falls

Subject-specific factors, such as body height, weight, mass distribution, and flexibility, play a crucial role in determining the dynamics of a fall and the resulting impact forces (97). Generic models often fail to account for this variability, leading to inaccuracies in predicting impact forces and assessing fracture risks. For instance, a taller individual falling sideways may experience distinct dynamics and higher impact forces compared to a shorter individual under similar conditions. This variability underscores the need for personalized modeling. Subject-specific dynamics modeling of falls provides a more accurate approach by integrating individual characteristics, such as body dimensions, weight distribution, and flexibility, which significantly influence the trajectory and forces of a fall. Such precision is essential for reliably predicting impact forces and evaluating fracture risk.

**Figure 4:**
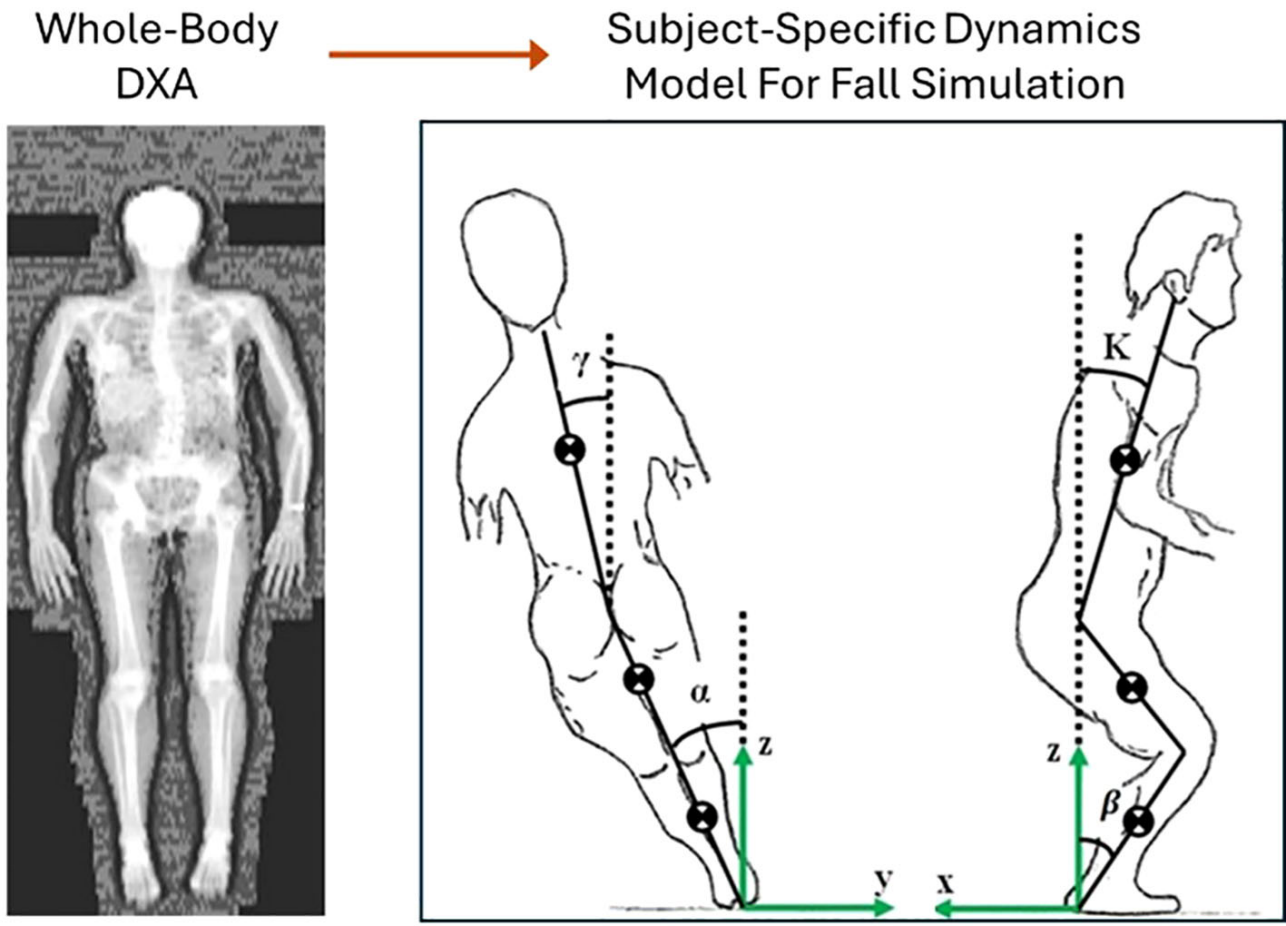
Construction of subject-specific dynamics model for simulating falls and predicting impact forces [modified from (26, 104)].

Whole-body medical imaging techniques, such as DXA or QCT scans, offer valuable data on bone geometry, body composition, and soft tissue distribution, which are key parameters for developing subject-specific dynamics models of falls. For example, DXA scans provide detailed estimates of regional fat and muscle distribution, as illustrated in **Figure 4**, which directly influence body mass properties and thus the dynamics of a fall. These parameters play a critical role in determining how the body interacts with the ground during impact and how forces are absorbed and transmitted through various tissues. By integrating this personalized information, subject-specific models can more accurately simulate fall mechanics, enhancing the prediction of impact forces and the evaluation of fracture risks. Substantial progress has been made in the development of subject-specific dynamics models for simulating falls. For instance, Luo et al. (25) developed and validated a method for constructing subject-specific dynamics models using whole-body DXA images. These models demonstrated improved accuracy in predicting impact forces during sideways falls, showing better agreement with experimental data compared to traditional empirical functions (98). Similarly, Fleps et al. (99) introduced a dynamic inertia-driven sideways fall protocol that tested full cadaveric femur-pelvis constructs under realistic fall conditions. This approach aimed to enhance the prediction of impact loads and fracture risk by replicating the dynamics of real-world falls, thereby bridging the gap between laboratory testing and clinical relevance. Studies using finite element models combined with dynamics simulations have demonstrated the potential to predict impact forces and their distribution during falls. For instance, researchers have utilized whole-body musculoskeletal models derived from DXA and QCT data to simulate falls and calculate site-specific impact forces (26, 44, 100). Some of these models have been validated using experimental data, such as motion capture systems and force plates, providing evidence of their predictive accuracy (25, 101). Furthermore, machine learning approaches have been integrated into fall dynamics modeling to enhance the efficiency and accuracy of simulations. Algorithms trained on large datasets can optimize model parameters, such as fall orientation and joint motion, based on subject-specific input (102). These approaches have improved the ability to predict real-world fall scenarios and their associated forces (103).

Dynamic fall models estimate forces that are subsequently used as boundary and loading conditions in finite element simulations. While these forces provide critical inputs, simplifications—such as assuming uniform force distribution or neglecting soft tissue effects—may introduce translational losses. These approximations can affect the accuracy of fracture risk predictions. Future research should focus on improving the fidelity of force translation and accounting for individual-specific factors to enhance prediction reliability.

### 4.2 Challenges in simulating real-world falls

Simulating real-world falls presents significant challenges due to the inherent complexity and variability of fall dynamics and subject-specific physiological factors (105, 106). Unlike controlled fall simulations, real-world falls are triggered by unpredictable and random events, such as tripping, slipping, or sudden loss of balance. These triggers introduce substantial variability in the initial conditions of the fall, including body posture, velocity, and the direction of movement at the onset of imbalance. Accurately replicating this randomness is crucial for realistic modeling but remains a significant hurdle.

Another critical factor is the reflexive response of muscles, which plays a pivotal role in influencing fall dynamics. When an individual loses balance, muscle reflexes are activated to counteract the fall, aiming to restore stability or reduce the severity of impact. Muscle activation affects joint stiffness (107, 108), a key parameter in modulating the ability of the body to respond to destabilizing forces (109). Increased joint stiffness, resulting from heightened muscle activation, can stabilize the joints, preventing excessive movement that might exacerbate the fall. Conversely, insufficient muscle activation or weak muscle may lead to joint instability, increasing the likelihood of an uncontrolled descent. In addition to joint stiffness, muscle activation directly influences the actuator force generated by muscle fibers (110), which determines the strength and speed of corrective movements. For example, in a sideways fall, the hip abductor muscles play a crucial role in resisting lateral displacement of the torso (111), while the quadriceps and hamstrings stabilize the knees to reduce the impact force upon ground contact (112). These coordinated muscle activations help control body posture and orientation during the descent, potentially shifting the impact away from vulnerable areas like the hip.

The timing and intensity of muscle reflexes also vary between individuals, influenced by factors such as age, neuromuscular coordination, and physical fitness. Older adults, for instance, often exhibit delayed reflex responses and weaker muscle activation (113–115), which compromise their ability to mitigate the effects of a fall. In contrast, younger and physically active individuals tend to have faster and stronger reflexes, enhancing their capacity to absorb and dissipate impact energy. Additionally, muscle activation patterns influence the redistribution of body mass during a fall (97). For instance, active engagement of the arms and legs can alter the center of mass trajectory, reducing the likelihood of a high-impact collision at critical sites such as the hip. However, excessive or uncoordinated muscle activation can lead to counterproductive effects, such as increased rotational forces or misaligned body segments, potentially exacerbating the impact at the end of the fall (116).

Incorporating the randomness of fall triggers and the variability in muscle reflex responses into fall simulations requires sophisticated modeling approaches, along with subject-specific physiological and biomechanical parameters, which are extremely challenging to characterize. Current methodologies often rely on simplified assumptions regarding initial conditions and reflexive actions, limiting their ability to represent the full complexity of real-world falls. Advanced techniques, such as stochastic modeling to simulate random fall triggers and neuromuscular modeling to replicate reflexive muscle responses, are necessary to address these limitations. Overcoming these challenges is critical for improving the accuracy and applicability of fall dynamics models in assessing fracture risk and developing personalized prevention strategies.

## 5 Conclusion and future outlook

Recent advancements in image-based approaches for hip fracture risk assessment have significantly improved our understanding of the interplay between bone strength and fall-induced impact forces. Finite element (FE) models derived from imaging modalities such as DXA and QCT enable individualized assessments of femoral strength by capturing bone material properties, microstructure, and geometry. These models mark a notable improvement over traditional statistical tools by incorporating patient-specific risk factors. Similarly, subject-specific dynamics modeling of falls has advanced the prediction of forces applied to the hip during real-world falls, offering the potential for more reliable fracture risk assessments. However, challenges remain in refining these approaches for improved accuracy and reliability.

Advancing biomechanical models for hip fracture risk assessment requires addressing several challenges:

- DXA-based FE models are limited by the projection of three-dimensional bone structures into two dimensions, which may introduce inaccuracies in estimating femoral strength. A significant limitation of DXA-based FE models is their sensitivity to body positioning during scanning, which can introduce variability in the estimated femoral strength and fracture risk. Ensuring consistent and accurate positioning is critical to improving the reliability of these models. QCT-based models offer greater anatomical detail but face barriers such as higher costs, increased radiation exposure, and limited accessibility. Both approaches require further improvements in accurately integrating material properties, such as bone density distribution and anisotropic strength, to enhance predictive accuracy.
- Characterizing bone mechanical properties based on medical images presents a significant challenge due to the difficulty of accurately mapping image-derived parameters, such as bone density, to mechanical properties like strength, stiffness, and toughness. Current methods often rely on empirical relationships that may not fully account for bone heterogeneity, anisotropy, and microstructural variations. To address these challenges, there is a need for more robust methodologies that couple advanced imaging techniques with experimental validation and multiscale modeling approaches, enabling more accurate prediction of mechanical behavior.
- In fall dynamics simulation, the complexity of real-world falls presents additional obstacles. Randomness in fall triggers, variability in fall trajectories, and reflexive muscle responses are difficult to replicate accurately. Current models often rely on simplified assumptions, limiting their ability to capture the variability observed in real-life scenarios. Advanced techniques, such as stochastic modeling for fall triggers and neuromuscular modeling for reflex responses, are needed to address these challenges and improve the reliability of impact force predictions.
- The integration of these image-based biomechanical models into clinical workflows remains limited due to technical and logistical constraints. Despite their detailed insights into hip fracture mechanisms, these models require further optimization for practical use in routine healthcare settings. Collaboration between engineers, clinicians, and imaging specialists is essential to bridge the gap between research and clinical practice.

In summary, image-based hip fracture risk assessment has made significant progress in offering patient-specific insights into fracture susceptibility. However, addressing technical challenges, refining modeling techniques, and facilitating clinical integration are critical for unlocking their full potential in improving fracture prevention and patient outcomes.
